# Prediction of Indian Summer-Monsoon Onset Variability: A Season in Advance

**DOI:** 10.1038/s41598-017-12594-y

**Published:** 2017-10-27

**Authors:** Maheswar Pradhan, A. Suryachandra Rao, Ankur Srivastava, Ashish Dakate, Kiran Salunke, K. S. Shameera

**Affiliations:** 1 Monsoon Mission, Indian Institute of Tropical Meteorology, Dr Homi Bhabha Road, Pashan, Pune, 411008 Maharashtra India; 2 Department of Atmospheric Sciences, Cochin University of Science and Technology, Kochi, Kerala 682022 India

## Abstract

Monsoon onset is an inherent transient phenomenon of Indian Summer Monsoon and it was never envisaged that this transience can be predicted at long lead times. Though onset is precipitous, its variability exhibits strong teleconnections with large scale forcing such as ENSO and IOD and hence may be predictable. Despite of the tremendous skill achieved by the state-of-the-art models in predicting such large scale processes, the prediction of monsoon onset variability by the models is still limited to just 2–3 weeks in advance. Using an objective definition of onset in a global coupled ocean-atmosphere model, it is shown that the skillful prediction of onset variability is feasible under seasonal prediction framework. The better representations/simulations of not only the large scale processes but also the synoptic and intraseasonal features during the evolution of monsoon onset are the comprehensions behind skillful simulation of monsoon onset variability. The changes observed in convection, tropospheric circulation and moisture availability prior to and after the onset are evidenced in model simulations, which resulted in high hit rate of early/delay in monsoon onset in the high resolution model.

## Introduction

The onset of Indian Summer Monsoon (ISM) over southern India popularly known as Monsoon Onset over Kerala (MoK), is regarded as the beginning of the rainy season in the Indian meteorological calendar^1^. Monsoon onset has an imprint over southern Peninsula with seasonal reversal of surface and upper atmospheric wind and convection associated with the built up of contrast in temperature and pressure between Indian continent and Indian Ocean during March to May^2^. Earlier studies^3,4^ have discussed the sudden changes in rainfall over central Pacific and Indian Ocean Kerala during onset. There is a steady increase in convection over southeast Arabian Sea (AS) which associates with cross-equatorial Findlater Jet or Low Level Jet (LLJ; ref.^5^). At south of Kerala over AS, monsoon westerlies strengthens in magnitude and vertical extent just before MoK^6^. The onset also involves the establishment of a low pressure region that forms over southeast AS and moves in a northerly direction towards Gujarat coast, which is popularly known as “monsoon onset vortex”^7^. Monsoon onset also involves steady buildup of moisture^8,9^ and kinetic energy^2^ over AS. The strengthening of south westerlies, enhanced moisture transport and development of strong convection over equatorial Bay of Bengal (BoB) convection region are observed prior to monsoon onset^10,11^.

Considering the above mentioned abrupt changes in various surface and upper air meteorological parameters, it appears that determination of summer monsoon onset is a tricky job. For a long time India Meteorological Department (IMD) used to declare monsoon onset date by observing abrupt temporal and spatial evolution various meteorological parameters like precipitation, winds and OLR^3^. This declaration process suffered from subjectivity as no specific quantitative thresholds were used^6^. Ref ^1^ ﻿reported MoK time series for years 1901 to 1980, where MoK is defined date of transition of rainfall beyond the threshold of 10 mm/day for first five days.

In most of the above mentioned methods onset declaration are limited to the thought that monsoon onset is a synoptic process. Monsoon onset was believed to be a localized synoptic process until studies like^4,9,12,13^ illustrated that the thermal, hydrological and circulations features during monsoon onset are influenced by large scale processes. Also it is shown that the monsoon onset is teleconnected to various global climate processes like ENSO, IOD, MJO etc.^5,12,14^. Therefore the methods which use parameters over a district to declare monsoon onset may not be suitable for forecasting monsoon onset at long leads because, they suffer bogus onset due to dominance of local phenomena and exclusion of large scale forcing^5,9,15^. Researchers have tried to determine and predict onset date using predictors depending on large scale circulation. Recently IMD has adopted objective definition of monsoon onset^16^ using an empirical tool based on regression of principal component of large scale circulation and precipitation. The onset date is being predicted at IMD at one month lead since 2005. The principal component regression model has a good skill of onset prediction with root mean square error (RMSE) of 4 days. A hydrological definition of monsoon onset can be obtained from^15^ where, monsoon onset index is defined by averaging and then normalizing vertically integrated moisture transport over a box in AS. Using this criterion, the onset dates were well captured, however withdrawal dates were too early. Ref.^17^, used an index formulated by averaging the zonal 850 hPa wind over southern AS (5°–15°N and 40°:80°E) and monsoon onset is defined as the day when the index crosses a threshold of 6.2 m/s. The defined threshold works quite well for the observation/reanalysis. But when predicting with models most of which has inherent biases, the threshold is not justifiable. In that case the zonal wind has to be bias corrected and a new threshold has to be set. ﻿Ref.^18,19^ have used meridional shear in 850 hPa zonal wind to define monsoon onset and withdrawal respectively. The meridional shear is computed by taking difference of zonal winds averaged over the 20°:30°N & 70°:90°E from zonal winds averaged over 5°:15°N & 70°:90°E. Since wind and moisture are better simulated than precipitation in the coupled general circulation models, these methods have an added advantage when used for operational forecasting monsoon onset^20^ evaluated onset creation based on integrated moisture and wind speed over AS and found that the criteria based on wind speed is simpler and more prone to bogus onset^21^ have shown the importance of lower level (800–900 hPa) temperature inversion over AS prior to monsoon onset, as it determines the moisture bearing capacity of the monsoon current and when it is destroyed by some synoptic event leads to rapid rising of moisture upwards resulting precipitation. Based on these observations^22^ defined monsoon onset in terms of zonal asymmetric temperature anomaly (temperature averaged over 10°:17.5°N; 65°:75°E and 850 to 600 hPa levels) and found the onset dates to be reasonably correlated with large scale SSTs over BoB and Pacific. The land-sea thermal contrast plays a major role in modulating the monsoon onset^23–25^ have reported the interaction of circulations due to heating of Tibetan Plateau and monsoon during the monsoon onset. During the late spring and early summer (May and June), the tropospheric temperature (200–500 hPa) over the Tibetan Plateau rises due to large release of the sensible heat from the elevated land. But during this time the north and south of Tibetan Plateau is comparatively cooler. The abrupt warming of the Plateau results in meridional temperature gradient over the regions south of it. The thermally induced vertical motion and the evolving monsoon system interact with each other to extend monsoon rainbelt to the northern parts^26,27^ objectively defined monsoon onset and withdrawal dates with the reversal of meridional tropospheric temperature gradient from negative to positive and from positive to negative respectively. Meridional temperature gradient is obtained as the difference of tropospheric temperature (air temperature averaged between 200 hPa to 600 hPa) between a northern box (40°:100°E, 5°:35°N) and a southern box (40°:100°E, 15°S:5°N) and is denoted by ∆TT. Accordingly monsoon onset date is defined as the date when ∆TT changes its sign from negative to positive. This definition of onset not only addresses the primary processes involved in monsoon onset with sudden development of organized convection, but is also suitable for dynamic forecasting of monsoon onset as temperature is well simulated by global models.

The early or delayed onset of monsoon can have serious impacts on agriculture and various other planning activities^28^. Hence a good long-lead forecast of the monsoon onset can greatly help in mitigating the adverse impacts of early or delayed onset. The predictability of monsoon onset is achievable by understanding the monsoon variability over different time and spatial scales. From interannual to interdecadal time scales ISM is linked with El-Nino and Southern Oscillation (ENSO^29,30^), Indian Ocean Dipole (IOD^31–33^), North Atlantic Oscillation^34^ and Pacific Decadal Oscillation^35^. DEMETER^36^ coupled models have shown moderate skills for seasonal hindcast^37^. Seasonal forecasts from ENSEMBLES^38^ multi model project have also shown better skill for interannual variability of ISM^35^ have shown that Coupled Forecast System (CFS) v2 at T126 (100 km) resolution has good seasonal skill (~0.5) in simulating ISM rainfall hindcasts initialized with initial conditions from February. Ramu *et al*. (2016) have reported improved skill (~0.55) of ISM rainfall prediction along with better interannual variability (~0.5) of ISMR with higher resolution CFSv2 (T382; 38 km). The better seasonal prediction skill in CFS-T382 is explained in terms of the improvements it has shown in SST, precipitation and wind patterns. The improvements in T382 as compared to T126 are discussed in detail in Ramu *et al*. (2016). They have reported that since the mean pattern in rainfall, SST and wind are better simulated in IITM-CFS, which leads to better seasonal prediction skill. The rainfall over Western Ghats and central Indian landmass are improved significantly which attributed to better representation of synoptic scale systems. Also the overestimation over eastern equatorial Indian Ocean and western Pacific are reduced considerably. The low level anticyclonic bias over Indian landmass has been reduced in IITM-CFS as compared to NCEP-CFS. In addition to this, the upper level (200 hPa) anticyclone over Tibetan Plateau and tropical easterly jet stream over Indian subcontinent are improved in IITM-CFS, which not only contributes to strength of monsoon but also are important during monsoon onset.

The present day developmental activities that converge towards the improvements and betterment of ISM prediction could not be considered complete without proving its skill for monsoon onset. Recently attempts have been made for prediction of monsoon onset at different temporal scales using state-of-the-art global dynamical coupled models^39^ have shown feasibility of monsoon onset prediction with Centro Euro-Mediterraneo sui Cambiamenti Climatici (CMCC) subseasonal forecasts initialized on 1^st^ May. Despite of having moderate seasonal prediction skill of ISM, the prior studies are limited to onset prediction at 2–3 weeks in advance and skill of predicting onset at long leads is still poor^40^ discuss operational onset prediction in Extended Range Prediction System. They have used three different objective criteria for the identification of onset. The indices defined for onset determination are based on precipitation, wind over west of Kerala and depth of low level westerlies. They have used a multimodel approach with ensembles from CFSv2 run at T126 and T382 horizontal resolutions and bias corrected SST is used to force the GFS. The real-time Extended Range Prediction System is able to predict onset with a RMSE of 3.6 days at a lead of 4-pentads. In the present study we will explore the predictability of monsoon onset at long leads (one season in advance) using state-of-the-art coupled dynamical prediction model (CFSv2) which has proven skill in simulating the synoptic (ref.^41^ under review), intra-seasonal^42^ and interannual^43,44^ time scales of monsoon.

## Results

### Observed Features

**Figure 1:**
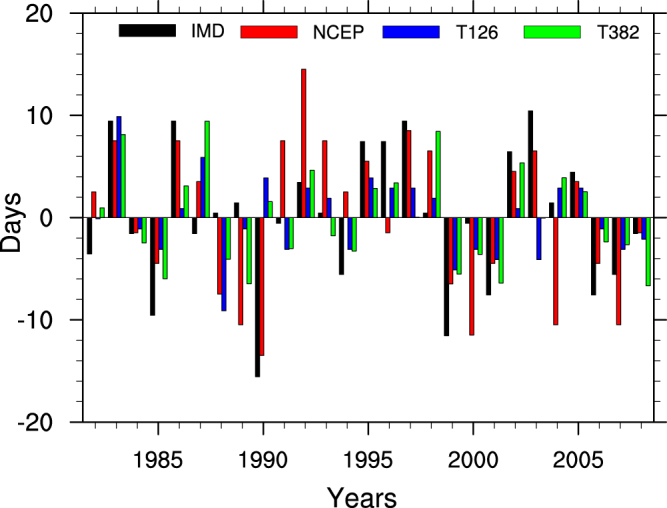
Time series (1982–2008) of monsoon onset date anomalies for observations (IMD, NCEP) and models (CFS-T382, CFS-T126). This Figure is created using NCAR Command Language (Version 6.3.0) [Software] (2015). Boulder, Colarado: UCAR/NCAR/CISL/TDD. http://dx.doi.org/10.5065/D6WD3XH5.

**Figure 2:**
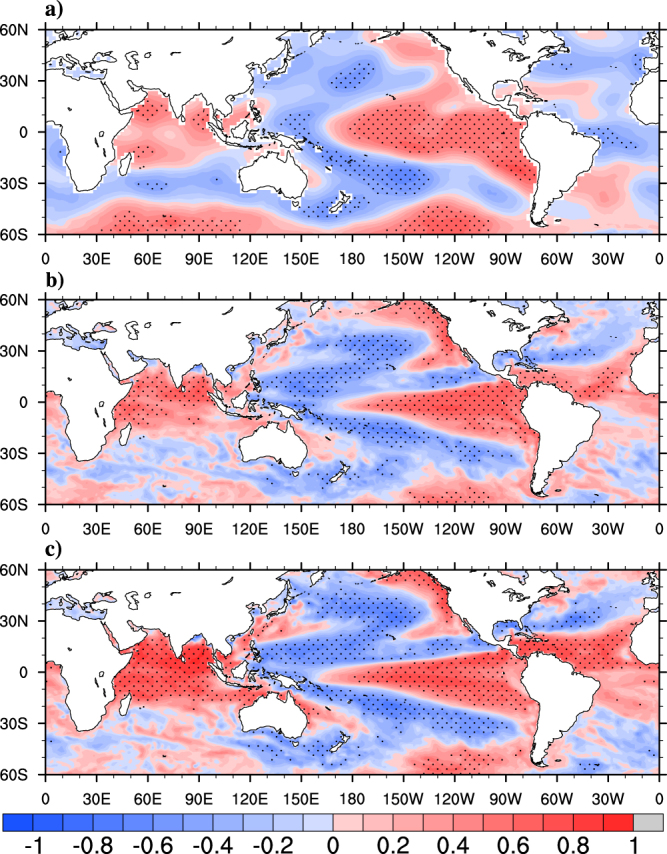
Correlation pattern between onset dates and mean SSTs during May-June (**a**) NCEP, (**b**) CFS-T126 (**c**) CFS-T382 for the period 1982–2008. The regions with correlation significant at 90% confidence level are shaded with black dots. This Figure is created using NCAR Command Language (Version 6.3.0) [Software] (2015). Boulder, Colarado: UCAR/NCAR/CISL/TDD. http://dx.doi.org/10.5065/D6WD3XH5.

The MoK derived from^16^ (hereafter will be called as IMD ODs) is compared with the onset dates based on thermodynamic (∆TT) criteria derived from National Centres for Environment Prediction (NCEP)- National Centre for Atmospheric Research (NCAR) reanalysis (hereafter will be called as “observed ODs”) for the period 1982–2008. As the thermodynamic onset marks the beginning of development of organized convection over Southern tip of India, a strong correlation^26,27^ of 0.61 (significant at 99% confidence level) between observed ODs and IMD ODs is found. The time series of IMD ODs, observed ODs for the period 1982–2008 is shown in the Fig. 1. In Table 1 the statistics of observed ODs and IMD ODs are presented. The mean onset based on ∆TT for the period 1982–2008 is 30^th^ May whereas that of IMD ODs is 2^nd^ June. The interannual variation in onset based on ∆TT (represented as standard deviation) is also found to be close to that of MoKs declared by IMD (both are approximately 7 days). The earliest and latest onset dates in NCEP are similar to that in IMD declared ODs. 1985, 1990, 1999, 2007 are the years when onset occurs as early as 5 days or more for both of the observations. Similarly 1983, 1986, 1995, 1997, 2003 are the years when onset occurs as delayed as 5 days or more. The delayed onset during 1983, 1995, 1997, 2003 are associated with El-Nino warming. But 1985, 1999 are the La-Nina years with early onset. As mentioned in earlier studies^23,26,45^ the monsoon onset is modulated by large scale forcing (such as air temperature over Tibetan high, Eurasia and Indian Ocean), it will be interesting to see how the monsoon onset is linked to global SSTs. Figure 2a shows the observed spatial correlation map between May-June mean SST and ODs. Significant positive correlations over central Pacific, eastern Pacific and Indian Ocean suggests that warmer SSTs over these regions are closely related to delayed onset, as discussed in ref.^14^. They have suggested a physical mechanism behind the positive correlations over central Pacific which says that, the warming over central Pacific results in an ascending branch (cyclonic circulation) of Walker circulation over central Pacific and a descending branch (anticyclonic circulation) over Indian landmass. Decrease in convection and latent heat release over Indian landmass associated with the subsidence cause decrease in tropospheric temperature over northern box and thereby cause delayed onset. Similarly warming over Indian Ocean modifies the regional Hadley circulation by increasing (decreasing) southern box (northern box) tropospheric temperature. Therefore warmer Indian Ocean SSTs are closely linked with delayed monsoon onset. El-Nino years are accompanied by warmer SST anomalies over central and eastern Pacific, whereas positive IOD events are accompanied by cooler SST anomalies over eastern Indian Ocean. Therefore monsoon onset gets altered during the El-Nino and IOD years through the modulation of Walker and Hadley circulations respectively caused by abnormal SST anomalies. By using ∆TT, the interannual variations in monsoon onset and its teleconnections with large scale processes can be captured quite satisfactorily as mentioned in ref.^14,27^. Hence, hereafter ∆TT is used as the index to determine ISM onset date.

**Table 1:** The statistics of onset dates for observations (IMD, NCEP) and models (CFST126, CFST382) for the period of 1982–2008.

Model/Obs	Mean	Earliest	Latest	STD	RMSE with IMD (with NCEP)
IMD	June-2	May-18	June-13	6.74	—
NCEP	May-30	May-18	June-14	7.4	6.44
T126	June-6	May-31	June-15	3.9	6.52(6.90)
T382	Jun-10	Jun-3	June-19	4.69	6.15(6.55)

**Figure 3:**
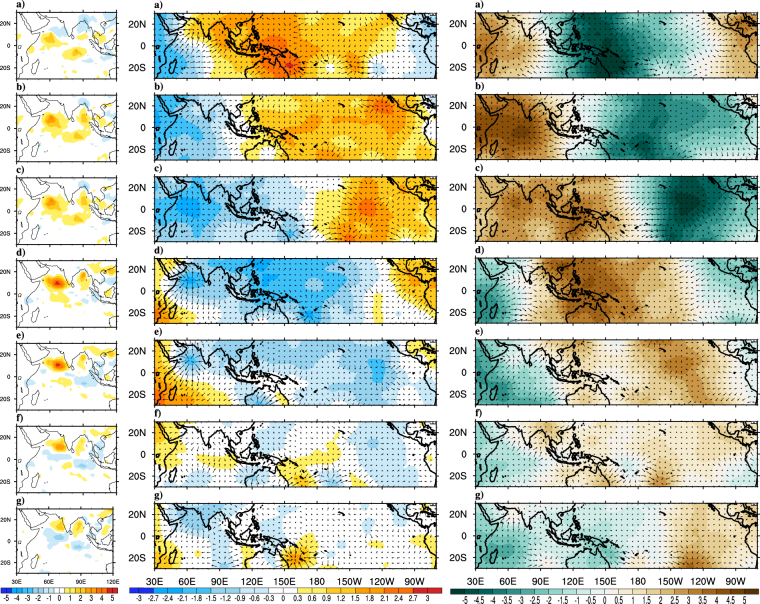
Composites of observed precipitation (Panel-I; mm day^−1^), VP200 (Panel-II; *10^6^m^2^ s^−1^) and divergent component of VIMT (Panel-III; *10^7﻿^kg m^−1^ s^−1^) anomalies for the period 1982–2008. Precipitation composites are at (**a**) Pentad −3, (**b**) Pentad −2, (**c**) Pentad −1, (**d**) Onset Pentad, (**e**) Pentad +1, (**f**) Pentad +2, (**g**) Pentad +3 and VP200 & VIMT composites are at (**a**) Lag-9, (**b**) Lag-6, (**c**) Lag-3, (**d**) Onset, (**e**) Lead-3, (**f**) Lead-6, (**g**) Lead-9. The magnitude of reference vector is 5 units for Panel-I, II. This Figure is created using NCAR Command Language (Version 6.3.0) [Software] (2015). Boulder, Colarado: UCAR/NCAR/CISL/TDD. http://dx.doi.org/10.5065/D6WD3XH5.

**Figure 4:**
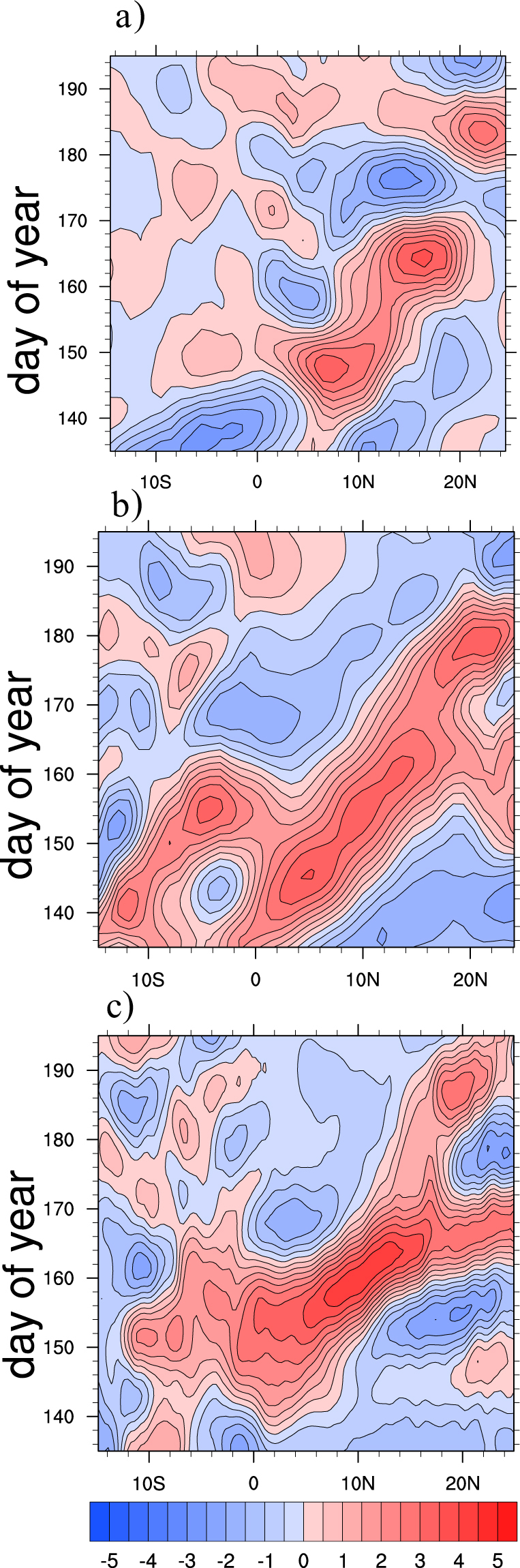
Latitudinal propagation of CISO anomalies (mm day^−1^) during 15^th^ May to 15^th^ July for (**a**) observations (**b**) CFS-T126 (**c**) CFS-T382. Time period for both of the model is 1982–2008 whereas, for observation time period is limited to 1997–2008. This Figure is created using NCAR Command Language (Version 6.3.0) [Software] (2015). Boulder, Colarado: UCAR/NCAR/CISL/TDD. http://dx.doi.org/10.5065/D6WD3XH5.

During the monsoon onset, some of the prominent large scale changes occur over Asian monsoon region are rapid increase of daily precipitation, vertically integrated moisture and kinetic energy at lower levels, strengthening of vertical shear in horizontal winds and setting up of a cyclonic vortex over AS^2,7^. In order to understand how the tropical atmospheric and oceanic features evolve during monsoon onset and how well the ∆TT criteria for onset is suited to capture these evolutions, composite analysis is carried out for various atmospheric and oceanic parameters. Figure 3I(a–g) show composites of rainfall anomalies from pentad −3 to pentad +3 with pentad 0 representing the onset pentad. At pentad −3 negative precipitation anomalies prevail over AS and BoB, which are in agreement with earlier reports by^5,6^. During pentad −2 to pentad −1 a band of positive rainfall anomalies (deep convection) is seen in east-west direction near southern tip of India^12^ have linked these anomalies to the positive convection anomalies that begin near the equator few pentads before the onset^6^ have reported these persistent and widespread rainfalls over Kerala and to its east and west are result of cyclonic vorticity to the north of fully developed LLJ. As the onset day approaches the positive anomalies gets intensified over north Indian Ocean, AS and BoB. On the onset date organized convective anomalies can be seen over AS and is known as monsoon onset vortex^7^. Post onset, the organized convection penetrates the Indian landmass causing monsoon rainfall over Western Ghats, East Coast and southern Indian landmass. Gradually the convection intensifies and area of maximum convection moves northward (more specifically northwest ward) due to inherent dominant 30–60 day intraseasonal oscillations^7,39,46^ which often accompanies an onset event. Also^47,48^ have pointed out the possible linkage between these northward propagating convection bands with the Climatological Intraseasonal Oscillation (CISO)s during the monsoon onset. Figure 4(a) shows the latitudinal propagation of CISO anomalies (obtained by averaging over longitudes of 55°E–75°E) around the observed climatological onset during May-June. The initial phase of the first episode of northward propagating CISO starts during late May or early June. Additionally it can be observed that the initial wet phase of CISO is associated with ISM onset as reported by^47,48^. Moreover studies like^6^ have used northward migration of ISO anomalies as one of the objective criteria to determine MoK.

The evolution of tropospheric circulation during the monsoon onset can be seen from the composite analysis of divergent component of horizontal wind at 200 hPa (VP200) and 850 hPa (VP850; not shown) as plotted in Fig. 3II(a–g). Centers of low (high) velocity potential are associated with divergent outflow (convergent inflow). Large scale positive VP200 anomalies that extend from BoB and eastern Indian Ocean to central Pacific can be seen during lag-9. At the same time negative VP200 anomalies persist over Africa and AS. As the time advances, the negative anomalies (upper level convergence and lower level divergence) over AS move northeastward and reach over Indian land mass, South China Sea, as discussed in ref.^6^. At lag-3 and monsoon onset date, confined upper (lower) level divergence (convergence) can be marked over southern AS, which can be related to the monsoon onset vortex^7^. A study by^49^ suggests that monsoon onset vortex builds up moisture and strengthening of westerlies over AS needed for setting up of MoK. The positive and negative VP200 anomalies show further eastward movement over Pacific. The eastward propagation is also evident at the lower levels. Therefore eastward propagating convergent (divergent) atmospheric waves at lower (upper) level are seen in coherence with the monsoon onset process. Another parameter that is used to investigate the monsoon onset phases is the vertically integrated moisture transport (VIMT). Figure 3III(a–g) shows the lag-lead composites of divergent (velocity potential) component of observed VIMT represented as shading and divergent component as vectors^15^ have tied the monsoon onset to the hydrologic processes (evaporation and precipitation) over AS and southern hemisphere. The moisture transport from the remote locations into the Indian monsoon region and hence the moisture availability plays an important role in initiating and sustaining monsoon through the adiabatic heating of the troposphere. Prior to onset, prominent divergence of moisture over western Pacific Ocean and prominent convergence over tropical Indian Ocean and Indian landmass are seen. During the onset phase the abundance of organized moisture convergence can be seen over north AS and tropical Indian Ocean that lead to monsoon onset over MoK. The transport of large quantities of moisture by the monsoon winds to the peninsular India is the reason for the buildup of moisture during the onset phase as reported in ref.^15^. Centers of deep convection near AS, BoB during the onset are also regions with strong moisture convergence and moisture is supplied from divergent regions of Southern Hemisphere and AS. The positive anomalies over Indian monsoon region also show eastward and northeastward propagation, which is discussed earlier for VP200 anomalies. After onset, the positive anomalies propagate eastward into the Pacific and Atlantic. Similarly negative anomalies propagate eastward into the Atlantic Ocean and reappear over Africa, India and Indian Ocean. Thus, VIMT composites shows strong agreement with the upper level wind circulation pattern discussed earlier. Both VIMT and wind composites suggest that during the onset phases, prominent eastward propagating MJO like phenomena prevails over the tropics. Both the circulation pattern and moisture transport features suggest the coherence between eastward propagating atmospheric Kelvin wave and onset phenomenon. Therefore monsoon onset has prominent relationship with MJO^50^.

### Model Simulations

From all of the analysis provided so far, it is demonstrated that monsoon onset is large scale phenomenon and is teleconnected to various tropical phenomena like ENSO & IOD and has close phase interaction with intrseasonal oscillations like MJO & CISO etc. Hence the onset variability can be predicted with a coupled global model, which has good skill in simulating tropical variability is attempted. In the present study, February IC seasonal hindcasts of CFS-T382 and CFS-T126 are considered for analyzing the prediction skill of onset variability and the associated physical processes. The time series along with the statistics of monsoon onset dates derived using ∆TT criteria for the model are presented in Fig. 1 and Table 1 respectively. CFS-T382 simulated mean onset date for the period 1982–2008 as June-10 and has a systematic bias of 11 days, whereas CFSv2 T126 shows bias of 7 days in mean monsoon onset date. Ref.﻿^43^ have shown that the T126 model has large negative bias in tropospheric temperature over Indian continent as well as Indian Ocean. The negative bias in tropospheric temperature has resulted in delayed climatological onset in T126. But T382 model has reduced negative bias over northern latitudes and enhanced positive bias over southern latitudes^43^. This caused the reduced tropospheric temperature gradient in T382 as observed and hence resulted in strong onset bias. The interannual variation of ODs in models (represented as standard deviation) is underestimated as compared to observation. The standard deviation (SD) in T382 (4.7) is found to be better than T126 (4.00). Root mean square error (RMSE) of ODs between T382 and observation (NCEP) is found to be 6.5 days whereas, between T126 and observation it is 6.9 days. The correlations between SST averaged for May-June and onset dates for model is plotted in Fig. 2c. Both the models capture the large scale teleconnections reasonably well with good correlations particularly over central Pacific and Indian Ocean. Considering the improved SD, RMSE of onset variability and its teleconnections in the high resolution model compared to low resolution model, the composite analysis and further discussions will be limited to high resolution model only, unless specified.

**Figure 5:**
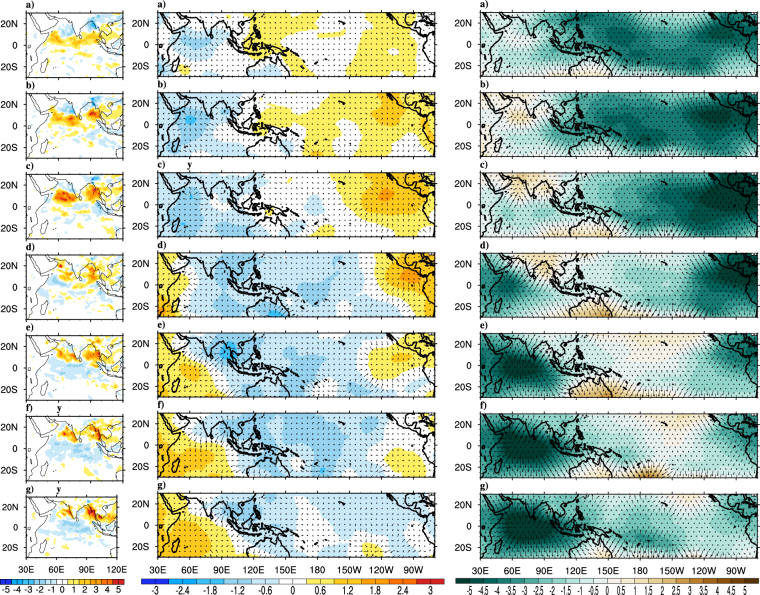
Composites of model (CFS-T382) simulated precipitation (Panel-I; mm day^−1^), VP200 (Panel-II; *10^6^m^2^ s^−1^) and divergent component of VIMT (Panel-III; *10^7^kg m^−1^ s^−1^) anomalies at (**a**) Lag-9, (**b**) Lag-6, (**c**) Lag-3, (**d**) Onset, (**e**) Lead-3, (**f**) Lead-6, (**g**) Lead-9 for the period 1982–2008. The magnitude of reference vector is 5 units for Panel-I, II. This Figure is created using NCAR Command Language (Version 6.3.0) [Software] (2015). Boulder, Colarado: UCAR/NCAR/CISL/TDD. http://dx.doi.org/10.5065/D6WD3XH5.

To investigate the CFST382 model’s fidelity to simulate the monsoon onset processes, the lead-lag composite analyses for various parameters are repeated, same as those which have already been discussed for observations. Figure 5I(h–n) shows the precipitation composites during lag-9 to lead-9 of onset date for the model. Before the monsoon onset, negative precipitation anomalies over northern AS and northern BoB and a band of positive anomalies over northern Indian Ocean can be seen. East-west extension of positive convective anomalies near southern tip of India due to fully developed LLJ is captured by model similar to observations. The model could also capture the confined positive rainfall anomalies over AS which are related to onset vortex during onset phase. The northward (more specifically northwestward) propagation of positive convective anomalies from the equatorial Indian Ocean to AS and along the Western Ghats is likewise well simulated by the model. The penetration of rainfall band into the Indian landmass during the post onset period causing monsoonal rainfall over Western Ghats, east coast and some interior parts of Indian landmass is furthermore captured well and is quite similar to observations. The phase locking between CISO and monsoon onset which was discussed earlier is also evident in model simulations (Fig. 4b). The first episode of CISO anomalies appear in the 1^st^ week of June in model simulations, which is little later as compared to observations where anomalies start two weeks prior to June. This delay is because of the inherent systematic bias in tropospheric temperature in the model which has been discussed earlier. Figure 5II(h–n) shows the composite analysis for 200 hPa horizontal wind velocity potential for the model from lag-9 to lead-9 days. Prior to onset, the large scale velocity potential field over Pacific and Indian Ocean has opposite sign, as noticed in observations. Consistent with the precipitation anomalies, confined upper level divergence over AS is seen even at lag-6 and lag-3 which is similar to formation of onset vortex in observations. The gradual eastward propagation of the divergent anomalies over Indian Ocean (and convergent anoamlies over Pacific) during lag-9 to lead-9 days due to the inherent atmospheric Kevin waves are satisfactorily reproduced in the model. The model simulated VIMT during monsoon onset process is shown in Fig. 5III(h–n). Prominent divergence of moisture over Pacific and convergence over AS and Indian landmass during the lag-6 to lag-3 period favors the monsoon onset. Interestingly, during this period, abundance of moisture over AS, which give rise to onset vortex can also be observed. The eastward propagation of positive anomalies from the Indian Ocean to the Pacific and the strengthening of negative anomalies over Indian Ocean after the monsoon onset can be seen, which are in agreement with VP200 anomalies. The composite analysis of precipitation, VP200, VIMT clearly shows that the model is able to capture the phase interaction between monsoon onset, CISO and global MJO. Therefore even at a lead of 3 months, the model simulations with respect to various known synoptic and large scale processes during monsoon onset are satisfactorily simulated with minor deviations in phase and magnitude.

Interestingly the high resolution model captures the large scale teleconnections of the monsoon onset reasonably well. One of the evidence is seen here as the teleconnection between OD and SST (Fig. 2), which establishes the Pacific and Indian Ocean’s role in modulating the monsoon onset. Furthermore the composite analysis (Figs 3 and 5) discussed earlier reveals that the changes in ocean-atmosphere during the onset are not just limited to Kerala coast; instead it has significant connection with the large scale circulation (VP200) and hydrological (precipitation and VIMT) changes. The results from model simulation show that the interannual variability, the teleconnections and the large scale changes in circulation and moist features are reasonably well simulated by the high resolution model.

### Early/Delay Onset

Since the model has reasonably captured the interannual variability of OD, it will be interesting to see the model’s performance to capture the extreme conditions i.e. early and delay in monsoon onset. This can be expressed as hit rate and penalty rate^51^. Hit rate is defined as the ratio between number of successfully simulated years and the total number of years. Similarly penalty rate is defined as ratio between number of unsuccessfully simulated years and the total number of years. Hit rate and penalty rate for early and delay in onset are shown in Table 2. Early years are defined as the years where onset date is less than climatological onset date minus 0.5*standard deviation of onset dates (OD < OD_climatology_ − 0.5* OD_standard deviation_). Similarly delay years are defined as the years where onset date is greater than climatological onset date plus 0.5*standard deviation of onset dates (OD < OD_climatology_ + 0.5* OD_standard deviation_). Out of ten years of early onset, model is successful for 7 years and failed for 3 years. Similarly for delayed onset the model is successful in 6 years out of 10 resulting hit rate equals to 60%. This implies early or delay in monsoon date is captured by the model with some degree of accuracy. The skillful representation of early or delay years by the high resolution model encouraged us further to repeat the composite analysis for the early or delay years to look at the reasons that favors model’s success. Therefore a comparative study between the observations and model simulations are carried out which includes composite of precipitation, VP200, VIMT during the early years.

**Table 2:** Early and Delay years in observations (NCEP) and their prediction in model (CFST382).

NCEP **Early** Years	CFST382 Predictions Whether captured (**Yes/No**)	NCEP Delay Years	CFST382 Predictions Whether captured (**Yes/No**)
**1985**	Yes	**1983**	Yes
**1988**	Yes	**1986**	Yes
**1989**	Yes	**1991**	No
**1990**	No	**1992**	Yes
**1999**	Yes	**1993**	No
**2000**	Yes	**1995**	Yes
**2001**	Yes	**1997**	No
**2004**	No	**1998**	Yes
**2006**	No	**2002**	Yes
**2007**	Yes	**2003**	No

**Figure 6:**
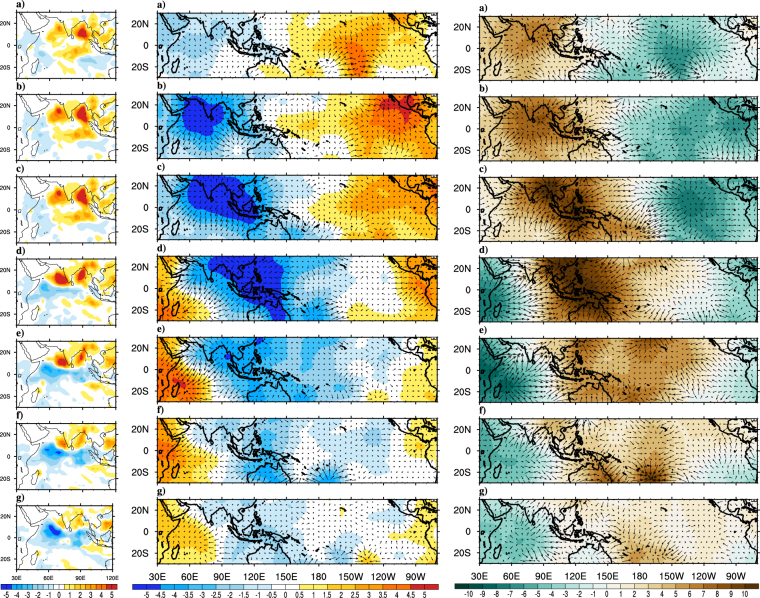
Composites of observed precipitation (Panel-I; mm day^−1^), VP200 (Panel-II; ﻿*10^6^﻿m^2^ s^−1^) and divergent component of VIMT (Panel-III; ﻿*10^7^﻿kg m^−1^ s^−1^) anomalies for the early years. Precipitation composites are at (**a**) Pentad −3, (**b**) Pentad −2, (**c**) Pentad −1, (**d**) Onset Pentad, (**e**) Pentad +1, (**f**) Pentad +2, (**g**) Pentad +3 and VP200 & VIMT composites are at (**a**) Lag-9, (**b**) Lag-6, (**c**) Lag-3, (**d**) Onset, (**e**) Lead-3, (**f**) Lead-6, (**g**) Lead-9. The magnitude of reference vector is 5 units for Panel-I, II. This Figure is created using NCAR Command Language (Version 6.3.0) [Software] (2015). Boulder, Colarado: UCAR/NCAR/CISL/TDD. http://dx.doi.org/10.5065/D6WD3XH5.

**Figure 7:**
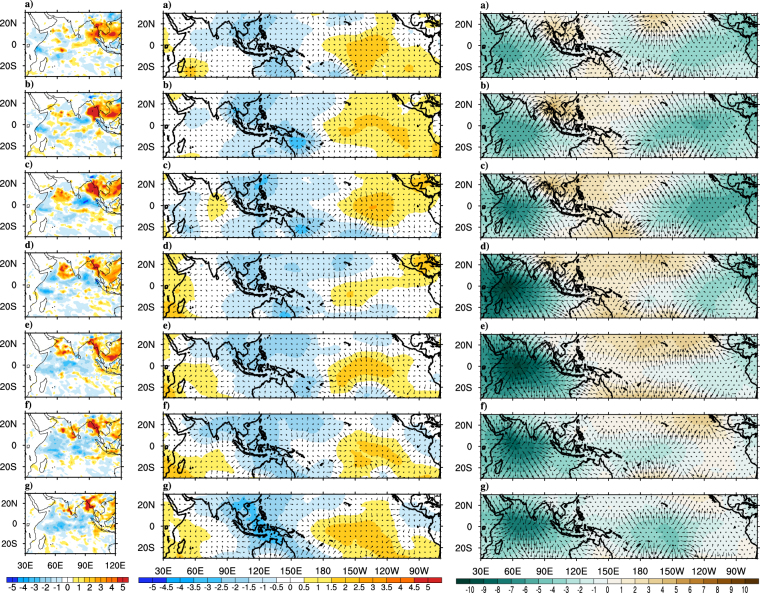
Composites of model (CFS-T382) simulated precipitation (Panel-I; mm day^−1^), VP200 (Panel-II; ﻿*10^6^﻿m^2^ s^−1^) and divergent component of VIMT (Panel-III; ﻿*10^7^kg m^−1^ s^−1^) anomalies at (**a**) Lag-9, (**b**) Lag-6, (**c**) Lag-3, (**d**) Onset, (**e**) Lead-3, (**f**) Lead-6, (**g**) Lead-9 for all the simulated early onset years. The magnitude of reference vector is 5 units for Panel-I, II. This Figure is created using NCAR Command Language (Version 6.3.0) [Software] (2015). Boulder, Colarado: UCAR/NCAR/CISL/TDD. http://dx.doi.org/10.5065/D6WD3XH5.

The observed precipitation, VP200 and VIMT composites for early onset years are shown in Fig. 6. Strong positive precipitation anomalies over AS, BoB and Indian landmass can be marked even 9 days prior to onset date, which is not the case when composites of all year are considered earlier. Strong upper level horizontal divergence (lower level convergence) is developed much earlier to onset date which favors the positive precipitation anomalies. Strong moisture convergence over Indian Ocean and Indian land mass gradually extending to western Pacific are also in agreement with the circulation and rainfall characteristics during pre-onset phase. As the time advances both the upper level divergence and moisture transport anomalies propagate eastward to the Pacific and anomalies while opposite sign appears over Indian Ocean. The composites show prominent existence of eastward propagating Kelvin waves (or MJO) even in early onset composites. The high resolution model successfully captures (Fig. 7), at long leads, positive rainfall anomalies over AS, BoB and Indian landmass and the ingress of precipitation anomalies from equatorial Indian Ocean to AS. The model is able to simulate the strong upper level divergence over India and Indian Ocean during the early years. The eastward propagation of wind divergence and moisture field are reasonably captured in the model; however, the propagation is slower as compared to observations. Both the upper level divergence and VIMT suggest a strong coherence of eastward propagating MJO like atmospheric phenomena with the Indian monsoon onset. Overall the large scale circulation and hydrological patterns during the early onset years are well simulated by the model^39^ have suggested the role of northward propagating ISOs in triggering the monsoon onset for some early years. Since T382 could capture the phase locking between CISO and onset quite well, therefore it has a reasonable capability to capture the early onset years.

The above results show that the IITM-CFS at high resolution has the capability to simulate the mean large scale evolution along with some of the synoptic features (like onset vortex). Moreover it has captured the remote influence of Pacific and Indian Ocean along with the interactions with intraseasonal fetures like MJO and CISO. The model’s inherent capability to reproduce the physical processes during the ISM onset raises the curiosity that, can the monsoon onset variability be predicted at 3 months lead using the dynamical model. The skills represented as anomaly correlation coefficient between observations and models (Table 3) suggest that the seasonal prediction model can be used for prediction of onset variability. CFSv2 T382 has a significant (at 99% confidence level) correlation with NCEP (0.49) as well as IMD (0.47). The skill in high resolution model is significantly higher than T126 (0.39 with NCEP; 0.37 with IMD). Considering present day seasonal prediction skill of various coupled global models, which is around ~0.5^37^, the prediction skill of ISM onset date variability is formidable. Therefore using the thermodynamic criteria, monsoon onset can be skillfully predicted 3 months in advance by IITM-CFS.

**Table 3:** Seasonal Prediction skills of onset dates represented as anomaly correlations between models (CFST126, CFST382) and observations (NCEP, IMD). Values significant at 99% confidence level are highlighted in bold.

SKILL	NCEP	T126	T382
**IMD**	**0.61**	0.37	**0.47**
**NCEP**	—	0.39	**0.49**

## Discussion and Conclusion

The internal dynamics and teleconnections related to ISM have been well studied and well modeled in earlier studies^52^. ISM onset is a process with sudden changes in rainfall, upper and lower level circulations, moisture availability, kinetic energy etc. Since onset is described as a complex transient feature of summer monsoon, the understanding of monsoon onset and its prediction from seasonal scale perspective has been lagging behind. Furthermore monsoon onset is an important aspect of decision making in various fields related to finance, agriculture, water management etc. The present study focuses on the fact that, monsoon onset is a large scale process and it can be simulated and hence predicted skillfully with modern state-of-the-art models (coupled models) and methods (objective criteria). The ΔTT criterion shows its capability in capturing monsoon onset reasonably well by having a good skill and interannual variation^26,27^. The observed teleconnections between ODs and SST have provided the justifications for the assumption that onset is a large scale process. Additionally the lead-lag composite analysis of precipitation, VP200, VIMT provides enough attestations for the effectiveness of ΔTT criteria. The abrupt changes associated with precipitation, upper and lower level circulations and moisture availability can be seen in the lead-lag composite analysis. Using this objective method, various well known synoptic (onset vortex, influence of ISOs) and large scale features (relations with Pacific and Indian Ocean, MJO) during monsoon onset are captured. Seeing the accomplishment of ΔTT criteria in capturing the observed features, it is engrossing to see its extension in models. IITM CFSv2 T382 is one of the highest resolution (~38 Km) models, which have shown very good skill (0.55, ref.^43^) for ISMR at seasonal time scales. It also has simulated the teleconnections better than its preceding low resolution model (NCEP CFSv2 T126). Therefore T382 is considered for testing the capability of the thermodynamic monsoon onset criteria. T382 has reasonable interannual variability of ODs as compared to observation. Model simulated teleconnections between ODs and SST are also close to observation. The predicted onset dates by fitting a multi regression equation between thermodynamical onsets and Indo-Pacific SST shows high correlations with the NINO 3.4 both for model and observations. The lead-lag composites similar to observation show that the model is able to capture the sequential evolution in large scale features during the onset. The composite analysis of precipitation, VP200, VIMT are able to reproduce the synoptic and large scale elements of onset as that of observation. The generation of convection anomalies near the equatorial Indian Ocean and their northward propagation to give rise to MoK are well evident in model as well as observations. The phase locking of 1^st^ episode of CISO and monsoon onset is also evident in model simulations similar to observations. The existence of eastward propagating atmospheric waves over tropical oceans during the onset phase can be seen in observations, which are also present in model simulations. The model also shows a good hit rate for both early (70%) and delay (60%) in onset, which is further reflected in composite analysis done for early onset years. Early development of the enhanced upper (lower) level horizontal wind divergence, convergence in vertically integrated moisture availability and positive precipitation anomaly are seen both in observation and model during the early onset years. The early onsets are related to the subsidence motion over equatorial Pacific and are influenced by eastward propagating atmospheric waves like MJO. The better simulation of physical processes during the onset, generate a skill of 0.49 and hit rate of 70% for simulation of early monsoon onset variability in the high resolution model. Therefore present study shows that, with suitable use of subjective criteria (thermodynamic criteria) and skillful model (IITM CFSv2 T382), monsoon onset is possible with a lead of 3 months in a seasonal prediction configuration, which will be quite helpful for the operational monsoon forecast community.

## Observation, Model and Methodology

In the present study all the analysis involving ISM onset are carried out for the period 1982 to 2008. Sea surface temperature data used is the Extended Reconstructed Sea Surface Temperature version 4 (ERSSTv4) taken from National Climatic Data Centre (NCDC), National Oceanic and Atmospheric Administration (NOAA), which has a resolution of 2° × 2°^53^. Daily gridded Observational rainfall over Indian landmass is obtained from the India Meteorological Department (IMD^54^) at 1° × 1° resolution for the mentioned period. For the analysis involving oceanic regions along with landmasses CPC (Climate Prediction Centre) Merged Analysis of Precipitation (CMAP^55^) pentad data set at a resolution of 2.5° and Global Precipitation Climatology Project (GPCP^56^) daily data at a resolution of 1° × 1° are used. CMAP data is available for the period 1982–2008 whereas, GPCP data is available for the period 1997–2008. Daily vertical profiles (1000 hPa to 300 hPa) of horizontal winds (zonal and meridional), specific humidity, the surface pressure and 200 hPa horizontal wind at a grid resolution 2.5° for the period 1982–2008 is obtained from National Centres for Environment Prediction (NCEP)- National Centre for Atmospheric Research (NCAR) reanalysis^57^.

In this study NCEP CFSv2-T126 along with IITM CFSv2-T382 hindcasts for the period 1982–2008 are used. NCEP CFSv2 is an improved version of CFSv1 (T62^58^) in terms of model resolution and physics^59^. On the other hand Indian Institute of Tropical Meteorology (IITM) CFSv2 is an improved version of NCEP CFSv2 in terms of model resolution. The high resolution model is the IITM-CFSv2 T382 run from 1 February to 25 February with 5 day interval each for a lead time of 9 months. Ensemble members are made from both 00 UTC and 12 UTC (i.e. total of 10 ensembles) and the hindcast period is from 1982–2008. The seasonal and extended range forecast of the ISM rainfall (ISMR) at the IITM, Pune is being carried out using this model since 2011. CFSv2 T382 is a fully coupled atmosphere-land-ocean model with atmospheric component as Global Forecast System (GFS^60^) and oceanic component as Geophysical Fluid Dynamics Laboratory Modular Ocean Model version 4p0d (MOM4^61^). The ocean component is a finite difference model at 0.25°–0.5° grid spacing. Additional NOAH land surface model^62^ and an interactive sea ice model^63^ is also constituents of the coupled model. The number of vertical layers in atmospheric component is 64 (sigma-pressure hybrid level) and that in oceanic component is 40 (height level).

The IMD onset dates are taken from^16^. The objective criteria used for determination of ISM onset is described in ref.^26^. Following the criteria, tropospheric temperature gradient (ΔTT) is computed as the difference in tropospheric temperature (defined as air temperature averaged between 200 hPa to 600hPa) of northern box (30°E–110°E and 5°N–35°N) and southern box (30°E–110°E and 15°S–5°N). The onset date is determined where ΔTT changes its sign from negative to positive. For both reanalysis and model, onset dates are calculated following the above criteria. The vertically integrated moisture transport (VIMT) used in the composite analysis is calculated using the expression^15^.

1 $$\mathrm{VIMT}=\underset{1000}{\overset{300}{\int }}\mathrm{qUdp}$$

where, q is the specific humidity, U is the horizontal vector wind, p is the surface pressure. The integration is performed from 1000 hPa to 300 hPa, as the contribution above 300 hPa is negligible. The velocity potential of VIMT (represents divergent component of moisture transport) is computed by taking inverse Laplacian of divergence in moisture transport^64^. To represent the circulation features, velocity potential of horizontal wind (200hpa) is computed by taking inverse Laplacian of velocity divergence at the same level. CISO is represented as 20–70 days filtered anomalies of daily precipitation climatology^47,48^. For the computation of CISO anomalies GPCP data is used, whereas for composite analysis CMAP data is considered.

### Data Availability Statement

Since the model source code is not open access and the hindcasts are run on experimental basis, the model data is not available as of now. All the observational data sets used are available on respective sites as mentioned in the manuscript.
